# Tumor microenvironment promotes prostate cancer cell dissemination via the Akt/mTOR pathway

**DOI:** 10.18632/oncotarget.24104

**Published:** 2018-01-09

**Authors:** Junlin Shi, Lihui Wang, Chunlin Zou, Yudui Xia, Siyuan Qin, Evan Keller, Atsushi Mizokami, Jian Zhang, Yi Lu

**Affiliations:** ^1^ Key Laboratory of Longevity and Aging-Related Diseases (Guangxi Medical University), Ministry of Education, Nanning, China; ^2^ Southern University of Science and Technology School of Medicine, Shenzhen, China; ^3^ Department of Pathology and Internal Medicine, University of Michigan, Ann Arbor, MI, USA; ^4^ Department of Urology, Graduate School of Medical Sciences, Kanazawa University, Kanazawa, Japan

**Keywords:** tumor microenvironment, prostate cancer, epithelial–mesenchymal transition, disseminated tumor cells, metastasis

## Abstract

Metastasis causes high mortality in various malignancies, including prostate cancer (PCa). Accumulating data has suggested that cancer cells spread from the primary tumor to distant sites at early stage, which is characterized by disseminated tumor cells (DTCs). However, lack of direct evidence of partial localized PCa cells occurring epithelial-to-mesenchymal transition (EMT) and disseminating to distant sites (e.g bone marrow). In this study, we used luciferase labeled PCa cells to establish an EMT mouse model and to detect whether DTCs spread into the bone marrow. We observed tumor cells existing in mouse bone marrow when tumor grew subcutaneously at palpable stage. Studies also showed that *ex vivo* tumor cells exhibited increased proliferative, migratory, invasive and angiogenesis abilities. When compared *ex vivo* tumor cells with parental cells, hallmarks of EMT including E-cadherin, Vimentin, Snail, and ZO-1 were altered significantly. Specifically, the *ex vivo* tumor cells showed more mesenchymal properties. Angiogenesis markers, including VEGFR2, VEGFR3, MCP-3, I-TAC, I309, uPAR and GROα, were also increased in the *ex vivo* tumor cells. Intriguingly, MCP-1 expression was dramatically increased in those cells. Mechanistic analyses indicated that AP1 mediates PCa EMT and the appearance of DTCs via the Akt/mTOR pathway. This study may provide potential therapeutic targets and diagnostic biomarkers of PCa progression and metastasis.

## INTRODUCTION

Prostate cancer (PCa) is the most common malignancy in men worldwide. In the United States, an estimated 161,360 new cases and 26,730 deaths were expected in 2017 [1]. Metastasis, one of the main characteristics of malignancy, contributes to the high mortality of cancer. Bone is a preferential site for the metastatic homing of PCa cells. Once PCa patients develop skeletal metastases, they eventually succumb to the disease; therefore, it is imperative to identify key molecular drivers of this process [2–5].

The epithelial-to-mesenchymal transition (EMT) is a biological process associated with marked changes in cell adhesion, polarity, and migratory properties. It is typically characterized by the upregulation of mesenchymal markers and downregulation of epithelial markers. Accumulating evidence from experimental and clinical studies suggests that the EMT process plays pivotal roles in tumor invasion and metastasis by endowing cells with a more motile, invasive phenotype [6–8].

During metastasis, single or small clusters of cancer cells spread from the primary tumor to distant sites. The tumor cells that spread to distant organs are characterized by disseminated tumor cells (DTCs), whereas those in the peripheral blood (PB) are circulating tumor cells (CTCs) [9]. Experiments showed that tumor cells that had undergone the EMT in cancer cell nests were able to invade into the vasculature, circulate and extravasate to distant organs such as bone marrow and form DTCs [10–12]. The presence of DTCs/CTCs may cause development of distant metastases, even after complete removal of the primary tumor. Increasing evidence suggests that the spread of tumor cells indicates metastatic disease and poor prognosis [13, 14]. In prostate cancer, Morgan et al. tested bone marrow aspirates from 569 men prior to radical prostatectomy (RP), and found DTCs in over 70% of patients’ marrow. Cohort analysis showed that DTCs were present in 57% of patients after RP with no evidence of disease (NED), and 86% of DTC-positive NED patients subsequently suffered biochemical recurrence. It was reported that NED patients with DTCs had a significantly greater risk of recurrence compared with patients without DTCs [15]. This evidence suggests that the presence of DTCs and CTCs is an early event in the tumorigenesis. Disseminated tumor cells may persist in a quiescent status in distant organs, like the bone marrow, but become activated again and grow into overt metastases when the microenvironment provides appropriate signals [16, 17]. The spread of cancer cells to distant organs represents a major clinical challenge in the treatment of cancer. Therefore, there is an urgent need to elucidate the mechanisms and clinical relevance of DTCs/CTCs in cancer progression.

Activation of the EMT plays essential roles in tumor cell dissemination and eventual metastasis [18]. However, there is a lack of direct evidence of the relationship between the EMT and DTC generation, and the molecular mechanisms behind any potential relationship remain unclear. In current study, we established an EMT mouse model and investigated whether DTCs spread into the bone marrow. Further, we explored the biological characteristics of tumor cell dissemination and proposed possible molecular roles for the EMT and DTCs in tumor progression and metastasis.

## RESULTS

### PCa cells undergo epithelial-mesenchymal transition and disseminate to bone marrow in xenograft mouse models

**Figure 1:**
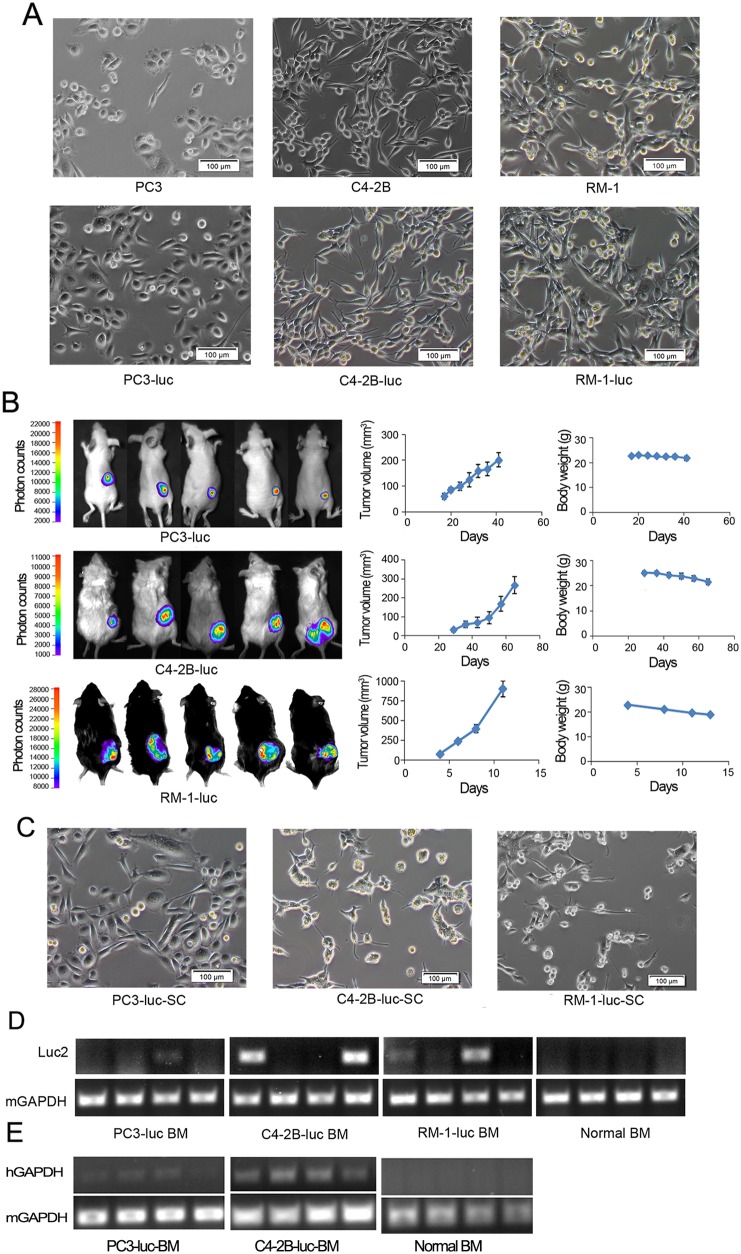
PCa cells undergo EMT and disseminate to bone marrow in mouse models **(A)** Morphology pictures of PCa cell lines, PC3, C4-2B and RM1 (upper panel), and the cells were transduced with luciferase reporter lentiviral systems, and named PC3-luc, C4-2B-luc and RM1-luc cells (lower panel). **(B)** PC3-luc, C4-2B-luc and RM1-luc cells were grown *in vivo* (n=10). The tumor volume and body weight were monitored. **(C)** Subcutaneous tumors were removed and *ex vivo* tumor re-cultured *in vitro*, named PC3-luc-SC, C4-2B-luc-SC and RM1-luc-SC. Compare to PC3 and PC3-luc, PC3-luc-SC became long spindle shape. **(D)** The mouse ilium, femur and tibia were collected when the experiment was done; bone marrow cells were flushed with PBS. Total RNA extracted from bone marrow cells were subjected to RT-PCR to determine whether subcutaneous tumor cells entered into bone marrow. *Luc2* gene represents tumor cell; mGAPDH used as internal control. **(E)** Total RNA extracted from bone marrow cells were subjected to RT-PCR to determine whether subcutaneous tumor cells entered into bone marrow. *hGAPDH* gene represents tumor cell; mGAPDH used as internal control.

Bioluminescence imaging (BLI) is a very popular and useful instrument for pre-clinical non-invasive *in vivo* studies. To use this system for the real-time observation of tumorigenesis and metastasis, a luciferase reporter lentiviral system was transduced into PC3 and C4-2B human PCa cells and RM1 murine PCa cells. There was no difference in the morphologic phenotypes and growth rate of PC3-luc, C4-2B-luc, and RM1-luc cells with their respective parental cells (Figure 1A). For *in vivo* experiments, PC3-luc cells were subcutaneously implanted into nude mice; C4-2B-luc cells were injected into CB.17 SCID mice, whereas RM1-luc cells were injected into C57BL/6J mice. The tumor volume was monitored using the BLI system and calipers twice a week (Figure 1B).

The mice were sacrificed at the endpoint. After sacrifice, the main organs of each mouse, including the lung, heart, liver, and kidney, were checked for metastatic foci; no metastatic tumors were found. Then, the xenograft tumors were harvested, and single cell suspensions were cultured with appropriate medium in a CO_2_ incubator. The *ex vivo* tumor cells were named PC3-luc-SC, C4-2B-luc-SC and RM1-luc-SC respectively. The morphology of the PC3-luc-SC, C4-2B-luc-SC and RM1-luc-SC cells had changed significantly compared with the cells growing *in vitro* (Figure 1C). To test whether the tumor cells had disseminated to the bone marrow, the ilium, femur and tibia were collected and bone marrow cells were flushed using PBS. Total RNA was extracted from the bone marrow cells and analyzed using RT-PCR. The results showed that part of mice expressed *luc2* in their bone marrow cells (Figure 1D). Further, we observed that most bone marrow samples from PC3 or C4-2B tumor-burden mice were detected human *GAPDH* expression (Figure 1E). These results suggest that some tumor cells have spread to the bone, which supports our hypothesis that tumor cells may move to distant organs at the tumor initiation stage.

### PC3 cells become more aggressive after growth *in vivo*

**Figure 2:**
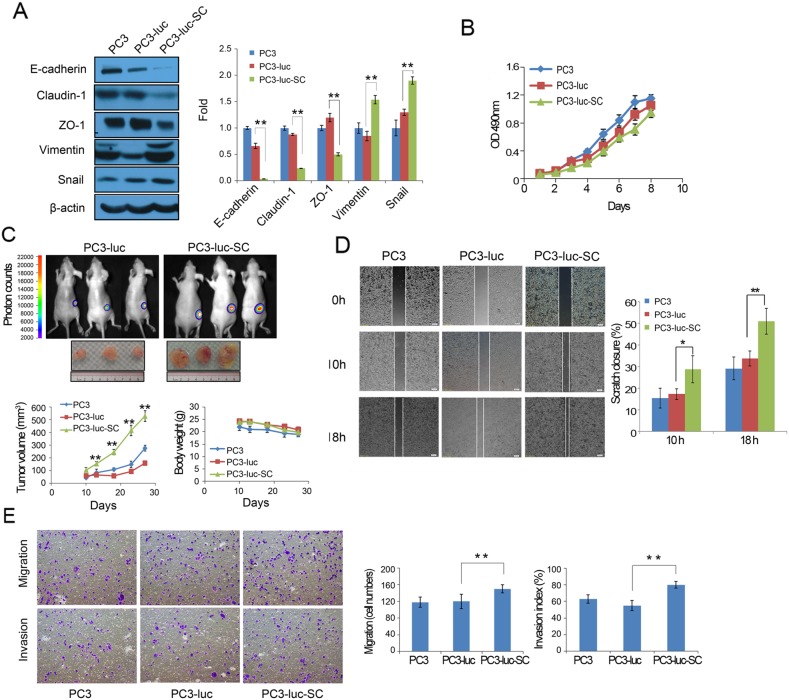
PC3-luc-SC cells occur to EMT and acquire greater abilities of proliferation, angiogenesis, migration and invasion *in vivo* **(A)** EMT markers (E-cadherin, Claudin-1, ZO-1, Vimentin and Snail) expressed in PC3, PC3-luc and PC3-luc-SC cells. The results were presented as mean±SE of three independent experiments. ^**^, *P*<0.01. **(B)** The proliferation of PC3, PC3-luc and PC-luc-SC cells *in vitro* were detected by MTS assay. **(C)** Upper panel: PC3-luc and PC3-luc-SC cells grown *in vivo* were monitored by bioluminescence imaging. Lower panel: Tumor volume of PC3, PC3-luc and PC3-luc-SC cells in xenograft model were measured by calipers. **(D)** Scratch wound healing assay showed the migration ability of PC3, PC3-luc and PC3-luc-SC cells. The results were presented as mean±SE of three independent experiments. ^*^, *P*<0.05, ^**^, *P*<0.01. **(E)** Transwell assay detected cell migration and invasion between PC3, PC3-luc and PC3-luc-SC cells. The results were presented as mean±SE of three independent experiments. ^**^, *P*<0.01.

We observed that the morphology of the *ex vivo* tumor cells changed significantly compared with the cells grown only *in vitro*. This suggests that something happens to cells passaged *in vivo* that results in morphological changes. To investigate what happens during tumor cell growth *in vivo*, the cell lysates of PC3, PC3-luc, and PC3-luc-SC cells were collected using standard procedures [19]. Western blotting showed that the expression of epithelial markers, such as E-cadherin, Claudin-1 and ZO-1, was significantly decreased in PC3-luc-SC cells, whereas the expression of mesenchymal markers such as Vimentin and Snail was increased compared with parental PC3-luc and PC3 cells (Figure 2A). These results indicate that PC3-luc-SC cells at least partially underwent the EMT in tumor microenvironment, acquired a mesenchymal phenotype, and perhaps spread to the mouse bone marrow. These data imply that prostate cancer bone metastasis is probably begun at the early growth of primary tumor.

However, *in vitro* cell proliferation assays showed that there were no significant differences between re-cultured cells PC3-luc-SC and PC3 or PC3-luc cells (Figure 2B). To further verify the changes in the biological characteristic of PC3-luc-SC cells using an *in vivo* model, PC3, PC3-luc, and PC3-luc-SC tumor cells were subcutaneously reinjected into nude mice. Compared with PC3 and PC3-luc cells, the subcutaneous tumors formed by PC3-luc-SC cells grew faster and more uniform *in vivo*. Blood vessels were more abundant on surface of PC3-luc-SC *ex vivo* tumors (Figure 2C). There was a remarkable difference in the growth rate of PC3-luc-SC cells *in vitro* and *in vivo*, which might provide an important evidence of microenvironment supporting tumor growth.

Wound healing and Transwell assays were used to investigate the difference in migration and invasion abilities of PC3, PC3-luc, and PC3-luc-SC cells. The results revealed that PC3-luc-SC cells exhibited significantly increased migration and invasion abilities compared with PC3-luc and PC3 cells (Figure 2D, 2E). Together, these results suggest that PC3-luc-SC cells become more aggressive after passaging *in vivo*.

### PC3-luc-SC cells produce excessive amounts of EMT-associated cytokines

**Figure 3:**
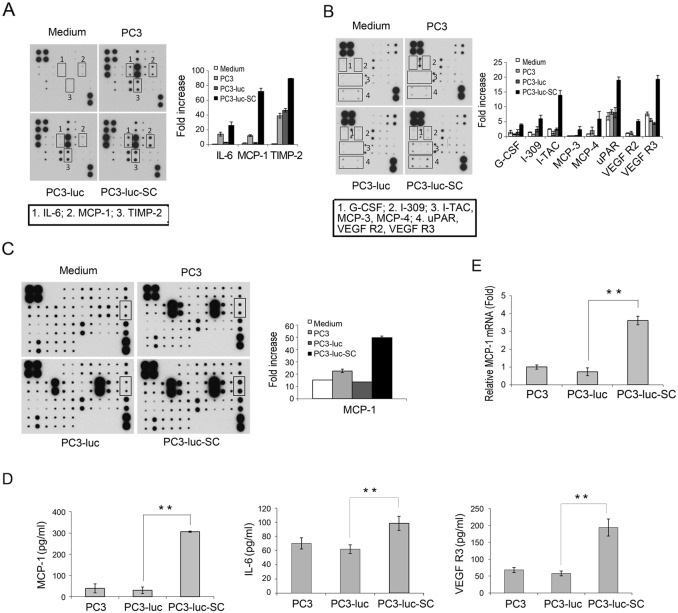
Angiogenesis and chemokine antibody arrays detect difference soluble factors in CM of PC3, PC3-luc and PC3-luc-SC cells, and validation by ELISA and qRT-PCR **(A and B)** Angiogenesis (1) and Angiogenesis (2) antibody array detected differentially expressed angiogenesis related factors in CMs from PC3, PC3-luc and PC3-luc-SC cells, blank medium used as control. **(C)** Chemokine antibody array detected difference expressed chemokines in CMs from PC3, PC3-luc and PC3-luc-SC cells, blank medium used as control. **(D)** The expression levels of MCP-1, IL-6 and VEGF R3 in CMs were verified by ELISA. **(E)** MCP-1 mRNA expression levels was measured by qRT-PCR. The results were presented as mean±SE of three independent experiments. ^**^, *P*<0.01.

The regrowth of PC3-luc-SC cells in a mouse model resulted in faster tumor growth, more uniform and with more vasculogenesis on the surface (Figure 2C). To identify changes of soluble factors in PC3-luc-SC cells, conditioned medium (CM) was collected from PC3, PC3-luc and PC3-luc-SC cell cultures, and the expression of angiogenesis-related factors and chemokines was measured using a Human Angiogenesis Antibody Array and Human Chemokine Antibody Array (RayBiotech). After normalization, the results showed that the expression levels of certain cytokines were upregulated in PC3-luc-SC cells, including interleukin-6 (IL-6), monocyte chemotactic protein-1 (MCP-1/CCL2), tissue inhibitor of metalloproteinases-2 (TIMP-2), granulocyte-colony stimulating factor (G-CSF), inflammatory factor (I-309, also called CCL1 [chemokine C-C motif ligand 1]), interferon-inducible T-cell alpha chemoattractant (I-TAC), MCP-3, MCP-4, urokinase-type plasminogen activator receptor (uPAR), receptor 2 for vascular endothelial growth factor (VEGFR2) and VEGFR3 (Figure 3A–3C).

To confirm the antibody array data, the levels of IL-6, MCP-1 and VEGFR3 were measured in the collected CM by ELISAs. Consistent with the arrays results, the levels of MCP-1, IL-6 and VEGF R3 were significantly higher in PC3-luc-SC cells compared with PC3-luc cells (Figure 3D). Moreover, qRT-PCR showed that *MCP-1* gene expression was greater in PC3-luc-SC cells (Figure 3E). These results are consistent with our previous findings [20, 21].

### Differential expression of transcription factors in PC3-luc-SC cells

**Figure 4:**
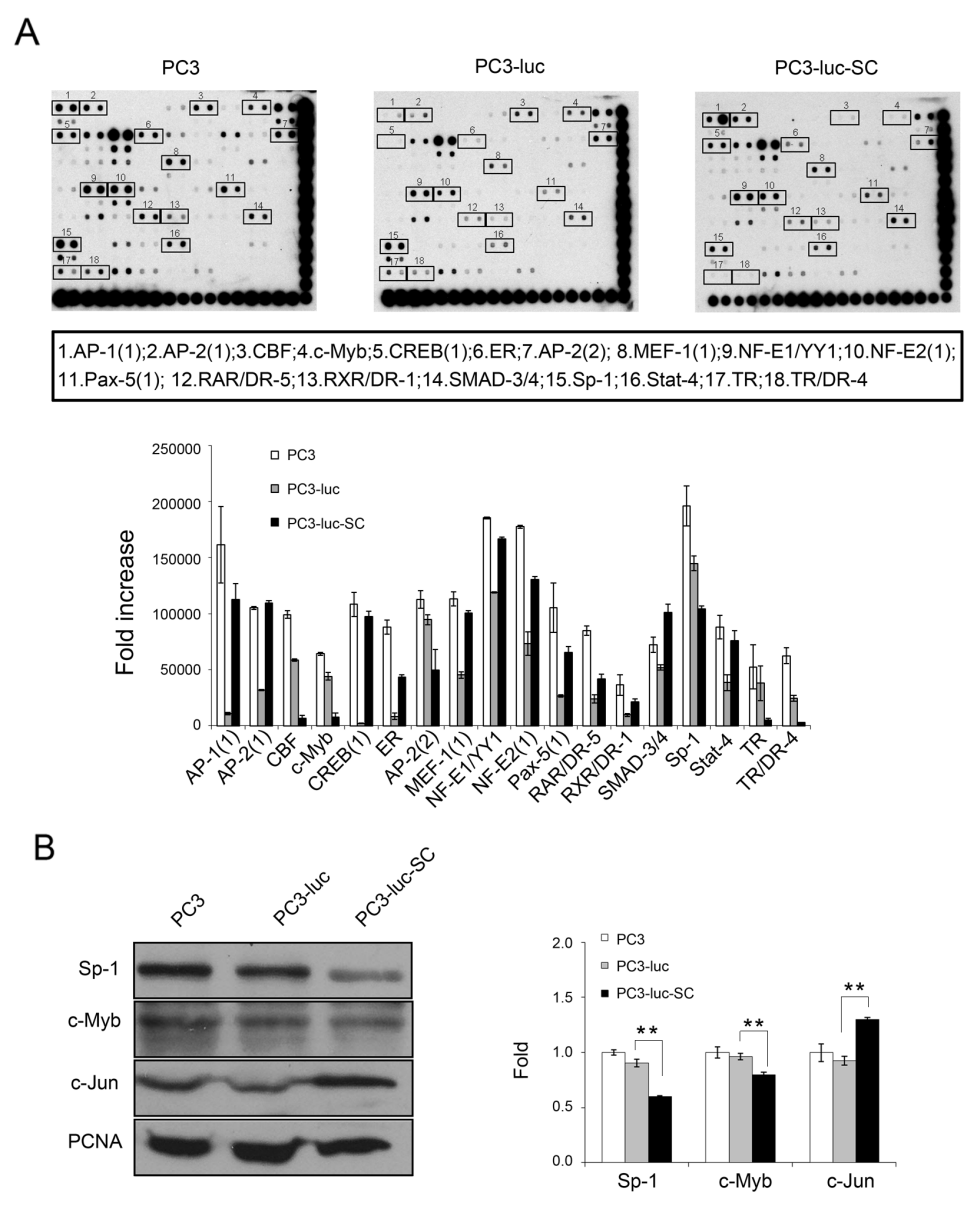
Differentially expression of transcription factors in PC3, PC3-luc and PC3-luc-SC cells, and verification by Western blotting **(A)** Protein/DNA array detected differentially expressed transcription factors between PC3, PC3-luc and PC3-luc-SC nuclear protein. **(B)** The protein levels of transcription factors, including Sp-1, c-Myb and c-Jun, were verified by western blotting assay. Compared to control cells, Sp-1 and c-Myb were downregulated, while c-Jun was upregulated in PC3-luc-SC cells. The results were presented as mean±SE of three independent experiments. ^**^, *P*<0.01.

To explore certain important transcriptional factors in PC3-luc cell growth *in vivo*, nuclear proteins were extracted from PC3, PC3-luc and PC3-luc-SC cells, and analyzed using protein/DNA arrays (Affymetrix) that included 56 transcriptional factors. The levels of several transcription factors, including activator protein 1 (AP-1), cAMP responsive element-binding protein 1 (CREB), estrogen receptor (ER), Myocyte enhancing factor 1 (MEF-1), nuclear factor (erythroid-derived 2) (NF-E2), paired box gene 5 (Pax-5), retinoic acid receptor/direct repeat element 5 (RAR/DR-5), retinoid X receptor/direct repeat element 1 (RXR/DR-1), MAD, mothers against decapentaplegic homolog 3/4 (SMAD3/4), and signal transducer and activator of transcription 4 (STAT-4), were increased significantly in the nuclei of PC3-luc-SC cells, whereas CAAT box General (CBF), Myb proto-oncogene protein (c-Myb), Sp1 transcription factor (Sp-1), thyroid hormone receptor (TR) and TR/DR-4 were decreased compare with PC3 and PC3-luc cells (Figure 4A).

Next, these data were confirmed by western blotting in the nuclear extracts used in the protein/DNA arrays. The results showed that c-Myb and Sp-1 were downregulated, whereas c-Jun was upregulated in PC3-luc-SC nuclei compared with PC3 and PC3-luc nuclei (Figure 4B). To our knowledge, this is the first study reporting a pivotal role of AP-1 in the biological behavioral changes of PC3 cells *in vivo* via the induction of chemokines and angiogenesis-related factors.

### PI3K-AKT signaling pathway activates the biological characteristics of PC3-luc-SC cells

**Figure 5:**
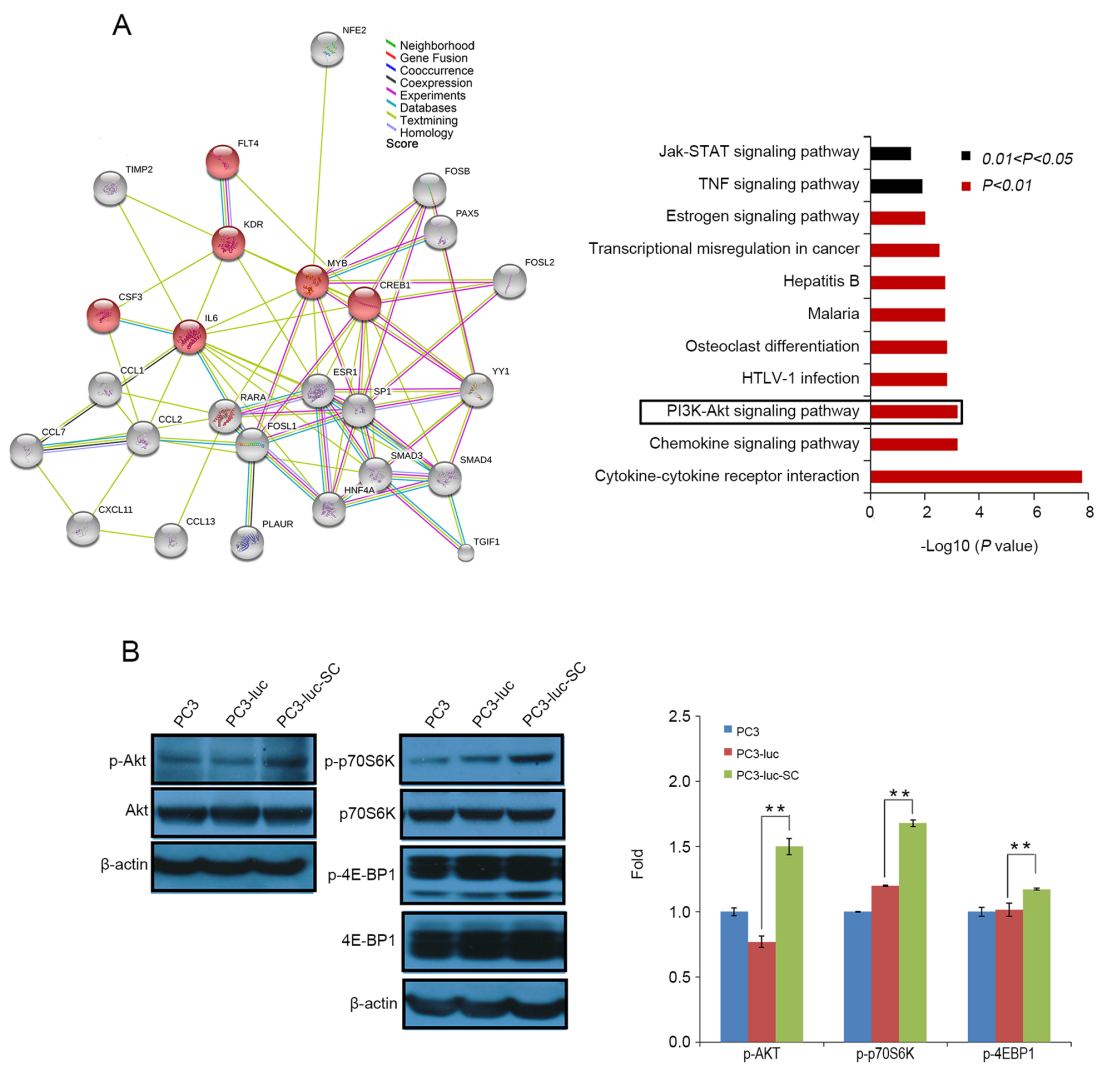
Protein networks promote tumor cells occurrence of EMT and generation of DTCs **(A)** STRING 10.0 analysis the differential expressed factors between PC3, PC3-luc and PC3-luc-SC cells from antibody array and protein/DNA array. PI3K-Akt signaling pathway has been found to activate PC3-luc-SC cells occurrence EMT and DTCs formation. **(B)** The activities of Akt, p70S6K and 4E-BP1 pathway were detected by western blotting. The results were presented as mean±SE of three independent experiments. ^**^, *P*<0.01.

STRING 10.0 (http://string-db.org/) analysis was used to predict protein-protein interactions based on the up- and downregulated factors identified in antibody arrays and protein/DNA arrays. The results suggested that the PI3K-Akt pathway may play fundamental roles in the behavioral changes of PC3 cell *in vivo* (Figure 5A).

Further, we confirmed these results using western blotting, which revealed that the levels of p-Akt (Ser 473) were increased in PC3-luc-SC cells. This suggests that the Akt signaling pathway is activated when the tumor cells growth *in vivo*. Intriguingly, p-p70S6K (T421) and p-4E-BP1 (Thr37/46) were also obviously increased (Figure 5B). These data suggest that in the tumor initiation stage, PC3-luc-SC cells activated the EMT process and spread to bone marrow via the Akt/ mTOR pathway.

**Figure 6:**
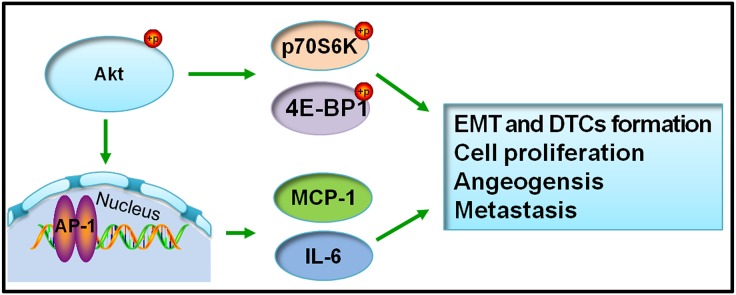
Schema chart of the molecular signaling pathway of biological characteristics changes of PC3 cells *in vivo*

In summary, Akt-related signaling pathways may play vital roles in the induction of the EMT in tumor cells and DTC formation in the early stage of PCa growth *in vivo*. On one hand, Akt phosphorylation promoted AP-1 activation and then induced the expression of chemokines such as MCP-1, IL-6, I-TAC and VEGF. This further led to tumor cell proliferation, angiogenesis and metastasis. On the other hand, activation of the Akt pathway increased the expression of p70S6K and 4E-BP1 to promote tumor cell proliferation, angiogenesis and metastasis (Figure 6).

## DISCUSSION

Cancer relapse and metastasis represent major clinical challenges in the treatment of cancer [22, 23]. Emerging evidence suggests that tumor cell EMT is an important prerequisite for the generation of CTCs and DTCs, which enables the dissemination of tumor cells into the circulation and surrounding tissues [16, 24, 25]. We hypothesized that there are protein networks in the tumor and its microenvironment that modulate the occurrence of the EMT and DTC generation. Blocking tumor cell EMT may benefit patients preventing potential metastatic risk. The early detection and analysis of DTCs and CTCs is very important to monitor and cease the development of overt metastatic disease.

In current study, we first used human PC3 and C4-2B xenograft mouse models and murine RM1 allograft mouse models to verify that tumor cells had partially undergone the EMT when they were grown *in vivo*. Indeed, tumor cells were detected in the bone marrow of mice carrying subcutaneous tumors, also known as DTCs; however, no overt organ metastasis was observed. This subcutaneous mouse PCa model provides direct evidence for the acquisition of EMT characteristics and invasion to the bone marrow.

Several lines of evidence suggest that the appearance of CTCs or DTCs has important clinical significance. For example, the presence of CTCs/DTCs can be used for prognostic evaluation [26, 27], to monitor tumor therapy [27], and as targets for tumor therapy [28] to reduce tumor recurrence and metastasis. The aim of this study was to find factors that regulate the generation and activation of DTCs, to seek novel approaches to reduce tumor recurrence and metastasis. In this study, PCa cells underwent the EMT in a mouse model, and tumor cells were detectable in the bone marrow of the mice (Figure 1D). Then, PC3-luc-SC cells were subcutaneously reinjected into nude mice. Experiments revealed that the tumor cells that had been passaged *in vivo* grew faster and had more abundant blood vessels on the tumor surface. These results are consistent with reports by other groups. Xia et al. reported that the induction of the EMT enhanced cell proliferation and invasion in lung cancer [29]. Fantozzi found that the EMT enhanced tumor cell angiogenesis by increasing the number of cancer stem cells [30].

Although PC3-luc-SC cell grew faster *in vivo* (Figure 2C), there was no significant difference *in vitro* proliferation studies compared with PC3 and PC3-luc cells (Figure 2B). This was dependent on autocrine factors, but paracrine factors derived from the microenvironment were also essential since both autocrine and paracrine factors promoted tumor cell growth and the EMT. Compared with PC3 and PC3-luc cells, PC3-luc-SC cells possessed more mesenchymal characteristics and increased migration and invasion abilities (Figure 2D–2E). Together, these suggest that tumor cell EMT and dissemination are synchronous with tumor growth *in vivo*. Further studies into this early event may elucidate the molecular mechanisms of tumor metastasis and achieve the goal of the early prevention of tumor metastasis.

By using antibody arrays, we identified that certain soluble proteins or factors, which related to the EMT, tumor proliferation, migration, invasion, angiogenesis and DTC formation, were expressed differentially. Specifically, we found that MCP-1 and IL-6 levels were dramatically increased in PC3-luc-SC cells compared with the control cells. MCP-1, also known as CCL2, is a chemotactic factor that plays important roles in recruitment and activation of monocytes during acute inflammation and cancer development. Several reports demonstrated that MCP-1 could induce the EMT [31, 32], and promote tumor cell proliferation [33], metastasis [34, 35], and angiogenesis [36–38]. Intriguingly, Fujisaki et al. reported that cancer-mediated adipose reversion promoted cancer cell migration via IL-6 and MCP-1 [39]. Chen et al. reported that the CCL2/CCR2 axis enhanced the IL-6-induced EMT [31]. In this study, we further observed that MCP-1 recruited macrophages to microenvironment (data not shown) and promoted tumor cell invasiveness, colonization and growth.

Analysis of signaling pathways in PC3, PC3-luc and PC3-luc-SC cells revealed that the accumulation of Akt phosphorylation could activate the transcription factor AP-1, which subsequently induced MCP-1 and IL-6 expression. More importantly, activated Akt could activate mTOR downstream genes p70S6K and 4E-BP1 to promote tumor cell proliferation, angiogenesis and metastasis. Several reports showed that c-Jun or c-Fos overexpression increased PCa cell proliferation and invasiveness [40, 41]. Although Thomsen et al. showed combined loss of *Junb* and *Pte*n in mouse model leads to invasive PCa [42]. Our results demonstrate that the Akt pathway plays a fundamental role in activating tumor cell EMT and dissemination. Regulating the Akt-related signaling pathway may reverse tumor cell EMT and DTC formation and further reduce tumor recurrence and metastasis.

In conclusion, the presence of DTCs is an early event in cancer pathogenesis. Tumor cell EMT is the first step of metastasis. In current study, for the first time we proposed that tumor cells obtain the mesenchymal phenotype in mouse models as well as an increased ability for cell proliferation, angiogenesis, migration and invasion. This process is activated by the Akt /mTOR pathway. However, additional studies are needed to fully understand the molecular mechanisms behind the occurrence of the EMT and DTC activation. Inhibitors of the Akt and related pathways could be applied to block the EMT process and DTC activation and thereby prevent tumor recurrence and metastasis.

## MATERIALS AND METHODS

### Cells and culture

Human PCa cells PC3 and human embryonic kidney 293T cells were obtained from the American Type Culture Collection (ATCC; Rockville, MD). Human PCa cells C4-2B derived from its parental LNCaP but with characteristics of skeletal metastasis were obtained from UroCor (Oklahoma City, OK). RM1 cells, a murine PCa cell line, were kindly provided by Dr. Evan Keller (University of Michigan Cancer Center, Ann Arbor, MI, USA). PC3 and RM-1 cells were cultured in RPMI 1640 medium (Invitrogen Co., Carlsbad, CA). 293T cells were maintained in Dulbecco’s Modified Eagle’s Medium (DMEM, Invitrogen). C4-2B cells were grown in T medium [21]. All media were supplemented with 10% FBS (Invitrogen) and 1% antibiotics (Invitrogen), maintained at 37°C, 5% CO_2_ in humidity air. All cell lines were genotyped for identity and were tested routinely for Mycoplasma contamination.

### Construction of luciferase reporter lentiviral system

The luciferase reporter lentiviral system was either purchased from GenePharma (Shanghai, China) or built by own lab. Lentiviral expression vector carrying luciferase was constructed. *Luc2* genes were amplified from pGL4.50 plasmid (Promega, Madison, WI) by PCR. Primers used for *Luc2* were: forward 5’-CACCATGGAAGATGCCAAAAACATTAAG-3’, reverse 5’-TTACACGGCGATCTTGCCGCCCTT-3’. The PCR products were inserted into pLenti6/V5 Directional Vector (Invitrogen). Then the recombinant construct was verified by sequencing (Invitrogen). The shuttle vector and packaging plasmids pRSV-REV, pRRE, pVSV-G (Promega) were transfected into 293T cells for assembly of lentiviral particles. The viral particles were collected after 48 hours transfection and were concentrated by ultracentrifugation.

### Transduction

Human PCa PC3 and C4-2B cells were plated in 24-well plates (5×10^4^ cells/well) overnight. The luciferase reporter lentiviral system (GenePharma, Shanghai, China) were diluted in 0.4ml (10^8^ TU/ml) complete medium containing 5μg/ml polybrene (Sigma, St. Louis, MO) and added to the cells for incubation for 24 hours at 37°C, followed by incubation in freshly complete medium for another 48 hours. Cell sorting collected strong GFP positive cells by Flow Cytometry.

RM1 cells were infected with the luciferase reporter lentiviral particles we built above in the presence of 5 μg/ml polybrene for 48 hours and positive clones were selected by 4μg/ml Blasticidin (Sigma) for at least 2 weeks.

PC3-luc, C4-2B-luc and RM1-luc cell lysates were collected and the luciferase activity assay was performed with Luciferase Assay System (Promega). Cell lysates and substrate of luciferase were added together in a microplate, the luciferase activity was recorded by luminometer reader (Glomax, Promega) and protein concentration of cell lysis was detected by BCA assay.

### Ethics statement

The animal experiments were conducted at the Center of Animal Research in Guangxi Medical University (Guangxi, China). The animal protocol was approved by the Institutional Animal Care and Use Committee, Guangxi Medical University. All the mice were housed under specific pathogen-free conditions in accordance with the NIH guidelines. The mice were kept in a temperature and humidity controlled environment under a 12 hours light-dark cycle and had free access to water and food at all time.

### Animal studies

Animal studies were used nude mice, SCID mice and C57BL/6J mice model respectively according to PCa cell types: PC3-luc, C4-2B-luc and RM1-luc cells. BALB/c nude mice were from the Center of Animal Research in Guangxi Medical University, CB.17. SCID mice and C57BL/6J mice were purchased from BEIJING HFK BIOSCIENCE (Beijing, China). Ten mice per group were used in these experiments (age: 4-6 weeks; male). Tumor cells were subcutaneously injected into the right flank of the indicated mice (2×10^6^ cells/mouse mixed with matrigel). Tumor size and body weight were measured twice a week and tumor volume was calculated using the formula as volume = length × width^2^/2 [43]. Tumor growth was monitored by BLI system weekly. Mice were injected intraperitoneal with D-luciferin (15 mg/ml, 10 μl/g) (Caliper, Hopkinton, MA) before imaging. They were anesthetized by 10% chloral hydrate intraperitoneal injection, and then luminescence imaging system for living animal (Lumazone, Roper Scientific, USA) was used for data acquisition. C57BL/6J mice were shaved and de-haired before imaging. Bioluminescence imaging was acquired with an exposure time of 120 seconds directly following administration of D-luciferin.

At the endpoint of experiments, mice were sacrificed and imersed in 75% alcohol; subcutaneous tumors were isolated and divided into 3 parts aseptically. One third of tumor was cut out and digested using 0.25% trypsin-EDTA in 37°C for one hour. Then single cell suspension was filtered by cell strainer and cultured in appropriate complete medium. These cells were *ex vivo* tumor cells. The others were kept in formalin or stored at −80°C. In addition, bone marrow cells were flushed by DPBS for total RNA extraction.

### RNA isolation and RT-PCR

Total RNA was extracted using TRIzol reagent (Invitrogen) according to the manufacture’s protocol. PCR primers for *Luc2* consisted of forward 5’-CAGCATGGGCATCAG-3’ and reverse 5’-ATGGGAAGTCACGAAGGT-3’; PCR primers for mouse *Glyceral-dehyde3-phosphatedehydrogenase (GAPDH)* consisted of forward 5’-AGGTTGTCTCCTGCGACTTCA-3’ and reverse 5’-GGGTGGTCCAGGGTTTCTTA-3’; PCR primers for human *GAPDH* consisted of forward 5’-AGCCACATCGCTCAGACA-3’ and reverse 5’-GCCCAATACGACCAAATCCA-3’. PCR products were subjected to electrophoresis on 1.5% agarose gel, stained with ethidium bromide [19].

### Western blottng analysis

Whole-cell lysates were prepared using standard procedures [19]. Nuclear extracts were prepared using Nuclear Extract Kit (Active Motif, Carlsbad, CA, USA) according to the manufacturer’s instruction. Briefly, whole cell lysates (50μg) and nuclear extract (20μg) were subjected to 10% SDS-PAGE gel and transferred to a PVDF membrane for Western blot analysis. The membranes were incubated with appropriate primary antibodies of E-cadherin, Claudin-1, ZO-1, Vimentin, Snail, c-JUN, SP-1, c-Myb, PCNA, p-AKT, AKT, p-p70S6K, p70S6K, p-4E-BP1, 4E-BP1 (Cell Signaling Technologies) and β-actin (Sigma) for overnight at 4°C. After being washed in TBST, the membranes were incubated with the secondary antibody conjugated with horseradish peroxides and the bands were detected using the enhanced chemiluminesence detection system (Thermo Scientific). B-actin and PCNA used as internal control for equal loading of whole cell lysate and nuclear extract respectively. Bands gray-scale was quantified using Photoshop and image J software.

### Cell proliferation assay

Cell proliferation assay was determined using the CellTiter 96 A_queous_ Non-Radioactive Cell Proliferation Assay Kit (Promega) according to the manufacturer’s instruction. Each individual experiment was performed at least three times independently using 6 wells for repeat.

### Wound healing assay

Wound healing assay was performed in triplicates in 6-well plates at high cell density (5×10^5^ cells/well). Twenty-four hours after cell seeding, cells were treated 3 hours with 20 μg/ml mitomycin. Scratches were gently and slowly introduced with a 200 μl pipette tip and cultures were replenished with serum-containing medium. Scratches were imaged at 0, 10, and 18 h after scratching. Wound closure areas were quantified using Photoshop and image J software.

### Transwell assay

Cells were seeded in 24-well matrigel invasion chamber (BD Biosciences, Bedford, MA), cell migration and invasion assays were done as previously described [44]. Invasive index was defined as the proportion of cells that penetrated the Matrigel-coated membrane to the number of cells that migrated through the uncoated membrane.

### Conditioned medium

Conditioned medium (CM) was harvested from PC3, PC3-luc and PC3-luc-SC cells for measurement of soluble factors [21].

### Antibody array

Human Angiogenesis Antibody Array C1000 and Human Chemokine Array C1 were purchased from RayBiotech (Norcross, GA, USA). Human Angiogenesis Antibody Array C1000 kit was consisted of 43 different antibodies spotted in duplicate onto two membranes. Human Chemokine Array C1 Kit was consisted of 38 different antibodies spotted in duplicate onto one membrane. Experiments were done as recommended by the manufacturer. Angiogenesis and Chemokine array were analyzed by imagine QTL software from GE and software from RayBiotech.

### ELISA

The levels of MCP-1, IL-6 and VEGF R3 in CM were measured with MCP-1 ELISA Kit purchased from R&D systems (Minneapolis, MN, USA), IL-6 ELISA Kit purchased from NeoBioscience (Shenzhen, China) and VEGF R3 ELISA Kit purchased from eBioscience (San Diego, CA, USA). ELISAs were performed according to the manufacturer’s protocols.

### Nuclear extract and protein/DNA array

Nuclear proteins of PC3, PC3-luc and PC3-luc-SC were extracted by Nuclear Extract Kit (Active Motif) according to the manufacturer’s instructions. The concentration of nuclear extract was quantified by ProStain^TM^ Protein Quantification Kit (Active Motif) in the light of the manufacturer’s instructions.

Protein/DNA assays were used to screen simultaneously a large number of transcriptional factors for DNA binding activity. Nuclear extracts were prepared as described above. The protein/DNA assays were carried out with the TranSignal TM Protein/DNA Array I (Panomics, Redwood City, CA, USA). Briefly, 8μg of total nuclear proteins were incubated with biotin-labeled DNA-binding probes (TranSignal Probe Mix) to allow the formation of protein-DNA (or TF-DNA) complexes. The protein-DNA complexes were separated from free probes by electrophoresis in agarose gels. The probes present in the complexes were eluted and hybridized to the TranSignal membrane dotted with correspond no labeled probes, and the signals were detected by exposure. Each spot was quantified by imagine QTL software from GE.

### Signaling pathway analysis

The functional association network of differentially expressed proteins from antibody array and protein/DNA array was constructed by STRING, a database of known and predicted interactions, including direct physical interactions and indirect functional interactions ((http://string-db.org/). And their potential signaling pathway has been given out.

### Statistical analysis

Statistical analysis was performed using Statview software (Abacus Concepts, Berkley, CA). Student *t* test (two tailed) was used to determine the significance of the *in vitro* cell proliferation, cell migration, cell invasion, western blotting analysis, ELISA, qPCR data between PC3-luc and PC3-luc-SC groups. PC3 and PC3-luc groups were used as control. Values are presented as the mean ± standard error (SE). All statistical analyses were performed using SPSS statistic package Version 16.0. Differences with a *P* < 0.05 were determined as statistically significant (^*^P < 0.05, ^**^P < 0.01).
